# Six Genes Associated with Lymphatic Metastasis in Colon Adenocarcinoma Linked to Prognostic Value and Tumor Immune Cell Infiltration

**DOI:** 10.1155/2022/4304361

**Published:** 2022-08-29

**Authors:** Baoquan Wang, Changjun Yin, Xu Yang, Huibo Shi, Zheng Zhang, Jun Zhou, Peitong Zhang

**Affiliations:** ^1^Department of Oncology, China Aacdemy of Chinese Medical Sciences Guang'anmen Hospital, Beijing 100053, China; ^2^Graduate School, Beijing University of Chinese Medicine, Beijing 100029, China; ^3^Department of Gastrointestinal Surgery, Sun Yat-Sen Memorial Hospital of Sun Yat-Sen University, Guangzhou 510120, China; ^4^Graduate School, Liaoning Technical University, Fuxin 123000, China; ^5^Department of Gastrointestinal Surgery, The Third Affiliated Hospital of Guangzhou Medical University, Guangzhou 510150, China

## Abstract

**Objective:**

The aim of the study is to explore the relationship between lymphatic metastasis genes, prognosis, and immune cell infiltration in patients with colon cancer.

**Methods:**

Based on the Cancer Genome Atlas Program (TCGA) database, differentially expressed genes and prognostic genes related to colon adenocarcinoma (COAD) lymphatic metastasis were screened and intersected. We used lasso and univariate Cox regression analysis to screen core genes and establish a preliminary prediction model. GO and KEGG enrichment analysis was used for lymphatic metastasis-related genes, and single GSEA was used for the final screening results. Finally, we evaluated the relationship between identified genes and immune cell infiltration.

**Results:**

A total of 1727 genes were differentially expressed between COAD patients with TNM stages of N0 and N1. After further screening, six core genes (RNU4-2, ZNF556, RNVU1-15, NSA2P6, RN7SL767P, and RN7SL473P) were obtained, and a preliminary prediction model was established, in which ZNF556 was a risk factor, and the rest were protective factors. Single GSEA showed that pathways such as systemic lupus erythematosus might play an important role in the initial lymphatic metastasis of COAD. GO and KEGG enrichment analysis of 1727 genes supported this result. Immune infiltration analysis showed that six genes were significantly correlated with T cell and NK cell families.

**Conclusion:**

Six core genes may affect COAD initial lymphatic metastasis through the systemic lupus erythematosus pathway and immune cell infiltration.

## 1. Introduction

Colorectal cancer (CRC) represents approximately 10% of all cancers and is the second most common cause of cancer deaths [1, 2]. Colon adenocarcinoma (COAD), one of the most common pathological types of CRC, has a high fatality rate worldwide [3]. Currently, the standard treatment for COAD is surgery combined with adjuvant chemotherapy or radiotherapy as per the clinical stages [4]. Patients with advanced COAD frequently cannot receive radical treatment because of distant metastasis, and their prognosis is very poor.

Lymphatic metastasis is a precursor of distant metastasis and a key determinant in the prognosis of patients [5]. Most solid tumors release growth factors such as vascular endothelial growth factor C (VEGF-C) to induce lymphatic vessel expansion (lymphangiogenesis) in primary tumors and in draining sentinel lymph nodes (LNs), thereby promoting LN metastasis [6]. It is worth noting that the occurrence of lymphatic metastasis is not accidental but a well-designed event, and the immune microenvironment may play an important role in this process [7].

For COAD, the potential molecular mechanism of lymphatic metastasis is still unknown; it is essential to explore its potential biomarkers. Immune cells in the tumor microenvironment participate in tumor cell lymph node metastasis through their complex interaction and biochemical function depth. Studies have shown that tumor-infiltrating lymphocytes (TILs) and CD57 play an important role in lymphatic metastasis of COAD by mediating local immune response and can also be used as independent prognostic factors [8]. Jianwei Lin's research was based on transcription factors (TFs) and established a prognostic risk model to predict the prognosis of patients with COAD and finally obtained a 7-gene prognostic model [9]. Appropriate biomarkers should effectively monitor disease progression and, to some extent, predict the patient's prognosis [10]. This study investigated the genes that affect initial lymphatic metastasis of COAD, speculated their mechanism, and discussed the relationship between them and immune infiltration to provide a new idea for clinical treatment of COAD lymphatic metastasis.

## 2. Materials and Methods

### 2.1. Data Access

This study used level 3 HT Seq-Counts format and HT Seq-FPKM format RNASeq data from the TCGA (https://portal.gdc.cancer.gov/) COAD project [11, 12], and we converted the data in fragments per kilobase per million (FPKM) format to transcripts per million (TPM) format. It comprised 521 COAD samples, with 41 from paracancerous tissues and 480 from tumor tissues. To verify the relationship between the gene expression and the immunophenoscore (IPS), we download the relevant information of COAD patients from The Cancer Immunome Atlas (TCIA) (https://tcia.at/home) [13, 14].

### 2.2. Differences in Gene Expression with Initial Lymphatic Metastasis

The clinical specimens with TNM stages of N0 and N1 were extracted and divided into two groups based on whether they had initial lymphatic metastasis or not.The purpose of this grouping is to explore the possible molecular mechanism of COAD initial lymphatic metastasis. The data format used in this part of the study is Counts. The differences in gene expression were analyzed by the DESeq2 package [15] of R software. The screening threshold was “adjusted *p*value <0.05 and log2 (fold change) > 1 or log2 (fold change) <−1.”

### 2.3. Genes Related to Prognosis

The survival package of R software was used for molecular screening of COAD prognosis [16]. In this part, we used data in the TPM format. The clinical information was retained after the paracancerous tissue group was removed from the data. Additional prognostic data were obtained from a Cell article [17]. “*p* cox <0.05” was taken as the screening threshold.

### 2.4. Enrichment Analysis

Gene Ontology (GO) enrichment analysis (BP: biological process; CC: cellular component; MF: molecular function) and Kyoto Encyclopedia of Genes and Genomes (KEGG) pathway analysis were performed on the selected differential lymphoid metastasis molecules [18–20]. “*q* value <0.2 and *p*. adj <0.1” were used as the threshold to enrich functional categories and pathways. The *R* software was used to examine the single GSEA of the six core genes [21, 22]. In terms of reference gene sets, we chose “c2 cp v7.2 symbols gmt.” “FDR (q value) < 0.25 and *p* adjust <0.05” were used as the threshold to filter pathways.

### 2.5. Screening of Genes and Establishment of the Prognostic Model

The selected prognosis and lymphatic metastasis-related genes were intersected. The intersection results were then screened using the “lasso regression methods” through the R package “glmnet” [23]. To assess the screening results of “lasso regression methods,” the Kaplan–Meier (K–M) survival curves were used to compare survival between low and high-risk groups using the survival package. Additionally, the time-dependent receiver operating characteristic (ROC) curve analysis (including 1-year, 3-year, and 5-year survival) was established to reflect the sensitivity and specificity of the results by the time ROC package [24]. We used univariate Cox regression analysis to further screen core genes [25]. For the corresponding variables of “*p* < 0.1,” multivariate Cox regression analysis was used to establish the model. The prognosis nomogram of the model results was drawn, and calibration analysis was performed to evaluate the actual prediction effect of the model [26].

### 2.6. Differential mRNA Expression of Genes and Its Relationship with Prognosis

The R software was used to validate the differential expression of six genes in different groups of COAD patients. The Kaplan–Meier survival curve of six genes was plotted by using a survival package [16]. Additional prognostic data were derived from an article of Liu's research [17]. The RNASeq data format of COAD patients used above is TPM.

### 2.7. Immune Infiltration Assessment

The immune cell infiltration of the obtained gene in COAD patients was evaluated by the ssGSEA algorithm [27]. We used the GSVA package in R software to complete this part of the research. The selected correlation analysis method is Spearman. The classification and description of specific immune cells can be found in Bindea's research [28]. The horizontal coordinate represents the gene, the vertical coordinate represents the immune cell, and the correlation coefficient is between −1 and 1. The difference was statistically significant when *p* < 0.05.

## 3. Results

### 3.1. Genes Screening and Results Evaluation

**Figure 1:**
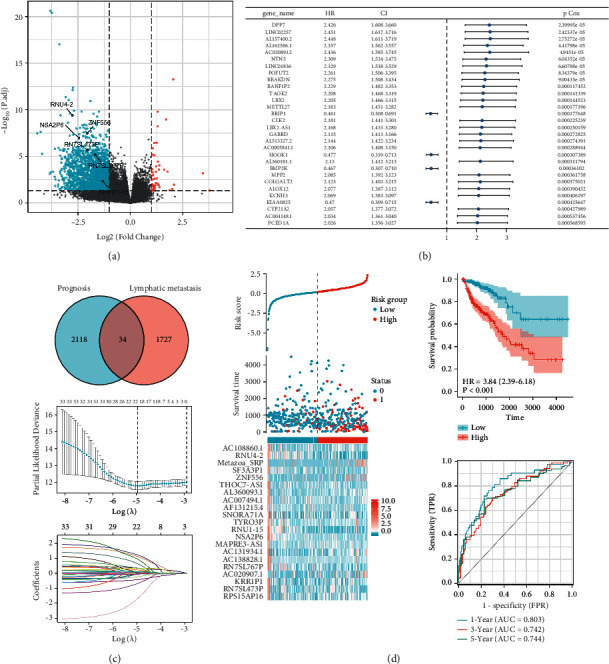
Screening of genes and evaluation of related results. (a) Volcano plot of lymphoid metastasis-related genes; (b) forest plot of prognosis-related genes only shows top 30; (c) Venn diagram and lasso regression method results; (d) lasso regression risk factor diagram, K–M survival curves, and ROC curve analysis.

A total of 1727 lymphatic metastasis-related genes (Figure 1(a)) and 2118 prognosis-related genes (Figure 1(b)) were screened. Among the genes related to initial lymphatic metastasis, 1682 genes were downregulated and 45 genes were upregulated. Among the prognosis-related genes, 1572 genes were caused by risk factors, while 546 genes were induced by protective factors. As shown in the Venn diagram, the intersection of the two types of genes yielded 34 genes. The variables were screened by lasso regression analysis, and the lambda.min was 21 (Figure 1(c)). Based on the results of the lasso regression analysis, the risk factor map was drawn, and the risk score, risk grouping, survival outcome, and 21 gene expression heat maps (Figure 1(d)) of COAD patients under lasso regression were obtained. The K–M curve and ROC curve (Figure 1(d)) were drawn based on the risk grouping of lasso regression results. There is a significant difference in prognosis between the high-risk and low-risk groups (*p* < 0.001, HR = 3.84). The ROC curve shows that the area under the curve (AUC) of the model obtained by lasso analysis for one year, three years, and five years is 0.803, 0.742, and 0.744, respectively, which has good prediction efficiency. It shows the reliability of lasso results.

### 3.2. GO and KEGG Enrichment Analysis of Initial Lymphatic Metastasis-Related Genes

**Figure 2:**
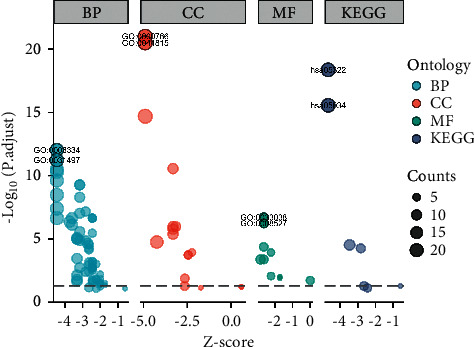
Bubble plot of lymphoid metastasis-related genes enrichment analysis.

**Figure 3:**
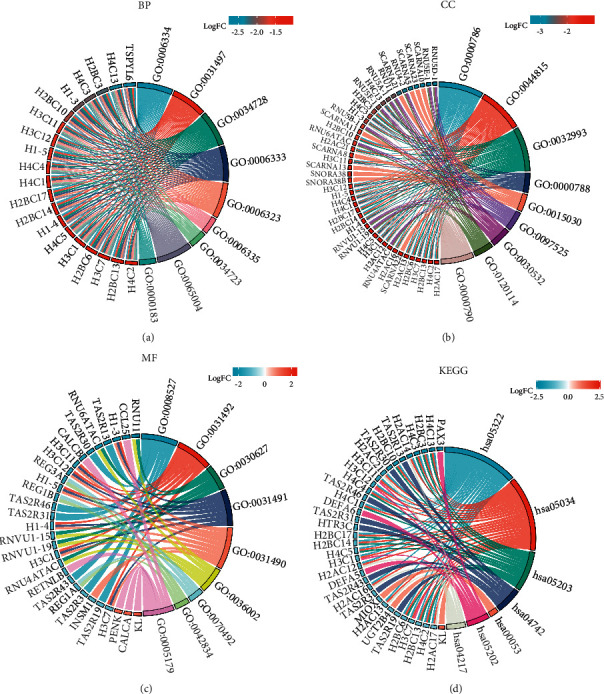
Circle plot of lymphoid metastasis-related genes GO and KEGG enrichment analysis. (a) BP: biological process; (b) CC: cellular component; (c) MF: molecular function; (d) KEGG: Kyoto Encyclopedia of Genes and Genomes.

A total of 1727 genes related to initial lymphatic metastasis were analyzed using GO and KEGG enrichment analysis to examine the possible molecular mechanism in the early stage of lymphatic metastasis in COAD patients. Under the condition of q value <0.2 and *p*.adj <0.1, there were 65 BP, 17 CC, 10 MF, and 7 KEGG (Figure 2). The main enrichment by BP analysis (Figure 3(a)) is nucleosome assembly, nucleosome organization, chromatin assembly or disassembly, DNA packaging, and protein-DNA complex assembly. This demonstrated that the biological process plays an important role in the initial lymphatic metastasis of COAD, and the results of CC and MF enrichment analysis supported this conclusion (Figures 3(b) and 3(c)). The main pathways enriched by KEGG analysis (Figure 3(d)) were systemic lupus erythematosus, alcoholism, and viral carcinogenesis. Furthermore, KEGG analysis revealed that transcriptional misregulation in cancer and necroptosis might be important in COAD lymph node metastasis.

### 3.3. Establishment of the Prognostic Model

**Figure 4:**
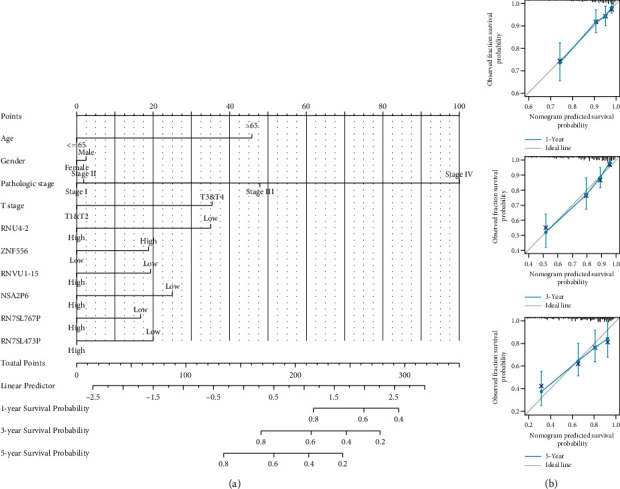
Nomogram for predicting and its evaluation. (a) Nomogram for predicting 1‐year, 3‐year, and 5-year OS in the entire TCGA cohort; (b) calibration curves of the nomogram on consistency between predicted and observed 1‐year, 3‐year, and 5-year survival in the entire TCGA cohort.

Genes obtained by lasso regression were analyzed by Cox regression analysis (used coxph function) to further screen core genes (Table 1). Univariate analysis showed that RNU4-2, ZNF556, RNVU1-15, NSA2P6, RN7SL767P, and RN7SL473P were significant and could be included in the multivariate regression model. Among them, HR < 1 of RNU4-2 and HR > 1 of ZNF556 were protective factors, and HR > 1 of RN7SL473P was a risk factor. Based on the results of multiple regression analysis of genes, the predictive map was formed by adding common clinicopathological factors (Figure 4(a)), and calibration analysis (Figure 4(b)) shows the results of the line chart. The advanced analysis parameters were as follows: several samples were recalculated in each group: 100, method: boot, data filtering: remove the normal group and retain clinical information. This part of the study aimed to develop a quantitative analysis tool that can predict the survival risk of individual patients. The nomogram calibration curve demonstrated that in the entire TCGA queue, when the index is one year, three years, and five years, the actual probability agrees with the model prediction probability.

### 3.4. Differential mRNA Expression of Genes and Its Relationship with Prognosis

**Figure 5:**
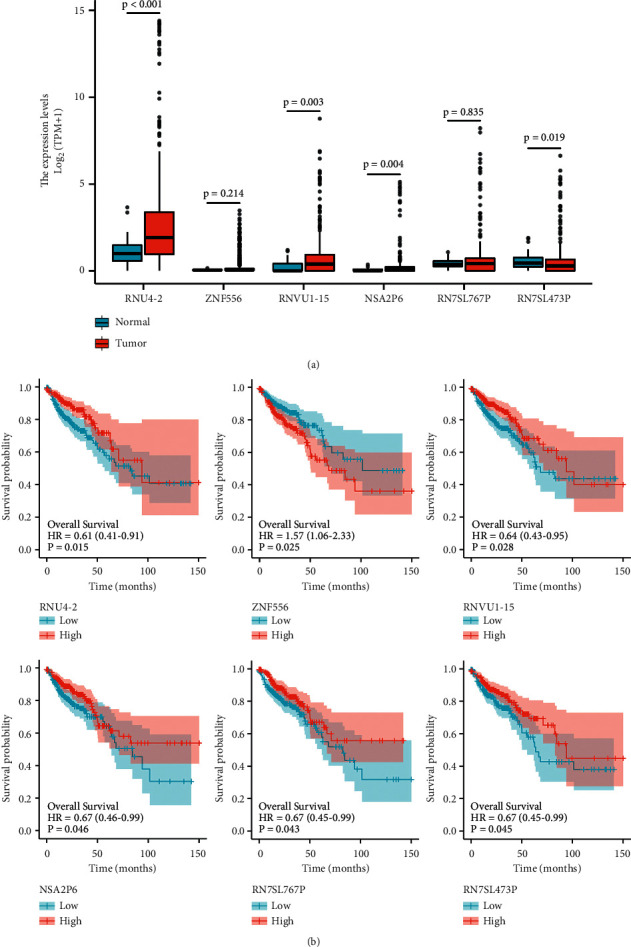
Differential mRNA expression of genes and its relationship with prognosis. (a) Box plot of differential expression of six genes in tumor tissues and normal tissues; (b) K–M survival curves between low and high expressions of each gene.

We used the TCGA database to confirm the differential expression of six genes in different grouping samples. It was discovered that there was a difference in the expression of mRNA between RNU4-2 and COAD tissues (Figure 5(a)). When the overall survival (OS) was used as an indicator, there were significant differences in the expression of six genes between survival patients and dead patients. When disease-specific survival (DSS) was used as an index, there were significant differences in gene expression except for ZNF556 (Supplement Figure 1). Moreover, the expression of six genes in different groups had significant differences in prognosis (Figure 5(b)). Amongthem, ZNF556 was found to be a risk factor, and the high expression group had a shorter survival time.Whereas the other genes were protective factors, and the high expression group had a longer survival time.

### 3.5. GSEA of Six Genes

**Figure 6:**
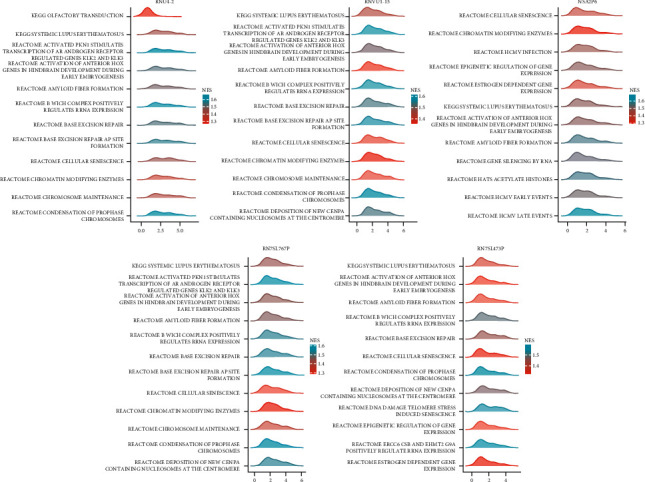
Mountain plot of GSEA enrichment analysis of six genes only shows top 12.

Single GSEA was performed on the six selected genes to investigate their possible functional pathways and mechanisms of action in COAD. ZNF556 was not enriched into the pathway that met the conditions, and the enrichment results of other genes were as follows (Figure 6). The findings revealed that “KEGG systemic lupus erythematosus” was significantly enriched in all five genes. This pathway plays an important role in the initial lymph node metastasis of COAD, and the previous GO and KEGG enrichment analysis results support this conclusion. Furthermore, we found “Reactome cellular senescence” “Reactome-activated pkn1 stimulates transcription of androgen receptor-regulated genes klk2 and klk3,” and “Reactome activation of anterior Hox genes in hindbrain development during early embryogenesis” played an important role in GSEA enrichment.

### 3.6. Immune Infiltration Assessment

**Figure 7:**
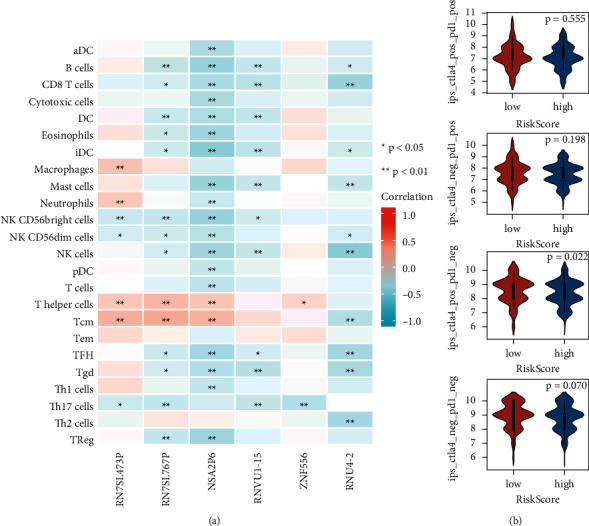
Six genes, respectively, associated with immune cell infiltration. (a) Correlation heat plot of immune infiltration; (b) correlations of six gene risk scores with IPS.

We investigated the relationship between six genes expression and the infiltration of 24 different types of immune cells (Figure 7(a)). It can be observed that there is a significant correlation between T cell and NK cell families and the expression of six genes. Previously, we discovered that the initial lymphatic metastasis of COAD may be highly related to the “KEGG systemic lupus erythematosus” pathway. Systemic lupus erythematosus is characterized by overactivation of the immune system, abnormal function of many immune cells, and the production of antibodies that attack their components [29–31]. These can significantly affect the tumor microenvironment. This conclusion was supported by immune cell infiltration analysis in COAD. Subsequently, we explored the relationship between the overall risk score of the six genes and IPS (Figure 7(b)). IPS is a good predictor of immunosuppressant response [32]. The immune checkpoints explored in this study include CTLA-4 and PD-1. Figure 7(b) shows the four types of IPS in TCIA: CTLA4 negative PD-1 negative, CTLA4 positive PD-1 negative, CTLA4 negative PD-1 positive, and CTLA4 positive PD-1 positive. The findings revealed a significant difference between high and low-risk scores in the “ctla4_positive_pd1_negative” group. This indicated that the overall risk score of the six genes may predict the response of anti-CTLA4 immunotherapy.

## 4. Discussion

Lymphatic metastasis plays a crucial role in tumor progression, enabling cancer cells to spread from primary tumors to distant organs [33]. Lymphatic metastasis is directly related to distant recurrence and prognosis in most tumors [34]. Moreover, the survival prognosis for N0 and N1 tumor patients has changed significantly, and even after surgical treatment, N1 patients also have a poor overall survival [35]. The molecular mechanism of lymphatic metastasis in COAD has not been thoroughly investigated. However, it is critical to investigate the molecular mechanism affecting the initial lymphatic metastasis of COAD and to explore biomarkers. Some scholars have examined the predictive value of miRNA in lymphatic metastasis and preliminarily determined miRNA that can predict lymphatic metastasis of colon cancer; however, the molecular mechanism has not been examined [36]. Based on the TCGA database, this study investigated the molecular mechanism affecting the initial lymphatic metastasis of COAD patients and preliminarily identified six core genes (RNU4-2, ZNF556, RNVU1-15, NSA2P6, RN7SL767P, and RN7SL473P).

ZNF556, as a colon cancer biomarker, has been demonstrated to possess a robust predictive ability, which validates the results of this study [37]. Unfortunately, ZNF556 was not enriched to the pathway in further the single GSEA in this study. RNU4-2 and RNVU1-15 are involved in RNA processing related to suicide and autism [38, 39]. This study revealed the potential of RNU4-2, RNVU1-15, NSA2P6, RN7SL767P, and RN7SL473P as biomarkers of COAD for the first time.

In GO enrichment analysis, cell division-related biological processes such as nucleosome assembly, nucleosome organization, chromatin assembly, or disassembly were found to be deeply involved in the initial lymphatic metastasis of COAD. This might be due to increased cancer cell division and the proliferation of new lymphatic vessels [40, 41]. KEGG enrichment analysis revealed that systemic lupus erythematosus was the most significantly enriched pathway, and furthermore, the single GSEA validated this result, suggesting that the systemic lupus erythematosus pathway may be crucial for initial lymphatic metastasis in COAD. According to relevant clinical studies, systemic lupus erythematosus enhances the risk for occurrence of various cancers and can lead to increased cancer-related mortality [42, 43]. However, its mechanism of action has not been elucidated. Systemic lupus erythematosus is an autoimmune disease that causes chronic multiorgan inflammatory damage and is characterized by the presence of nuclear autoantibodies, leading to the formation of autoimmune complexes, which are further deposited throughout the tissue, causing chronic inflammatory lesions. Its chronic inflammation might have a role in apoptosis, immunosuppression, or activation by influencing the tumor microenvironment, consequently affecting the occurrence and progression of tumors. The elucidation of the exact mechanism can be used as the direction of further research.

According to the findings of the above single GSEA enrichment analysis, immune infiltration in the tumor microenvironment may play a role in the initial lymphatic metastasis of COAD. As a result, this study examined the relationship between 24 different types of immune cells and the expression of six genes. The results revealed that the expression of these six genes was significantly associated with T cell and NK cell families. Further investigation revealed that IPS scores of patients also differed between different groups of six-gene risk scores. Similar studies have selected IRF1 as a biomarker to explore its relationship with immune cell infiltration and COAD metastasis [44]. IRF1 is associated with metastasis and the degree of immune infiltration of CD8^+^ T cells (general), dendritic cells, T-helper 1 cells, and T cell exhaustion in COAD, further demonstrating that immune cell infiltration can affect COAD lymphatic metastasis. Combined with this study, it can be seen that the above process is closely related to the expression of these six genes, although the specific mechanism remains to be explored.

In summary, based on the TCGA database, this study investigated the genes associated with the initial lymphatic metastasis of COAD and their mechanisms and initially established a predictive model. Finally, six core genes were obtained, and systemic lupus erythematosus was considered to play a significant role as its action pathway. Moreover, immune infiltration assessment showed that these six genes may promote COAD lymphatic metastasis by influencing immune cell infiltration. These provide potential targets for immunotherapy to prevent COAD development.

However, there are some unavoidable limitations in our research. As the study is based on bioinformatics analysis, there are no in vivo or in vitro experiments verifying the conclusions of this study. The research on the mechanism has not been thoroughly explained and verified. These should be further investigated for improvement.

## Figures and Tables

**Table 1:** Univariate and multivariate Cox regression analysis results.

Characteristics	Total (*N*)	Univariate analysis		Multivariate analysis	
		Hazard ratio (95% CI)	*p* value	Hazard ratio (95% CI)	*p* value
AC108860.1	462	0.975 (0.768–1.238)	0.836		
RNU4-2	462	0.879 (0.792–0.977)	0.017	0.920 (0.806–1.051)	0.220
Metazoa_SRP	462	2.088 (0.669–6.522)	0.205		
SF3A3P1	462	0.885 (0.565–1.386)	0.593		
ZNF556	462	1.384 (1.079–1.775)	0.010	1.440 (1.119–1.853)	0.005
THOC7-AS1	462	0.686 (0.404–1.163)	0.161		
AL360093.1	462	1.015 (0.689–1.494)	0.941		
AC007494.1	462	0.907 (0.607–1.356)	0.635		
AF131215.4	462	0.614 (0.327–1.154)	0.130		
SNORA71 A	462	0.998 (0.895–1.113)	0.970		
TYRO3P	462	0.864 (0.428–1.743)	0.683		
RNVU1-15	462	0.732 (0.559–0.957)	0.023	0.788 (0.563–1.102)	0.163
NSA2P6	462	0.505 (0.225–1.134)	0.098	0.672 (0.233–1.940)	0.462
MAPRE3-AS1	462	0.937 (0.528–1.660)	0.823		
AC131934.1	462	1.139 (0.856–1.515)	0.373		
AL138828.1	462	0.577 (0.217–1.537)	0.271		
RN7SL767P	462	0.770 (0.571–1.039)	0.088	1.063 (0.713–1.586)	0.765
AC020907.1	462	1.166 (0.920–1.478)	0.203		
KRR1P1	462	1.011 (0.828–1.235)	0.913		
RN7SL473P	462	0.778 (0.577–1.048)	0.098	1.030 (0.675–1.572)	0.890
RPS15AP16	462	0.978 (0.765–1.251)	0.858		

## Data Availability

The data supporting the findings of this study are available from the corresponding author upon request.

## Supplementary Materials

Supplementary Materials Supplement Figure 1: expression of six genes in different groups.
