# Supplementary figures and images for: Six Genes Associated with Lymphatic Metastasis in Colon Adenocarcinoma Linked to Prognostic Value and Tumor Immune Cell Infiltration
Source: Evid Based Complement Alternat Med. 2022 Aug 29;2022:4304361. doi: 10.1155/2022/4304361 (PMC9444393; doi:10.1155/2022/4304361)

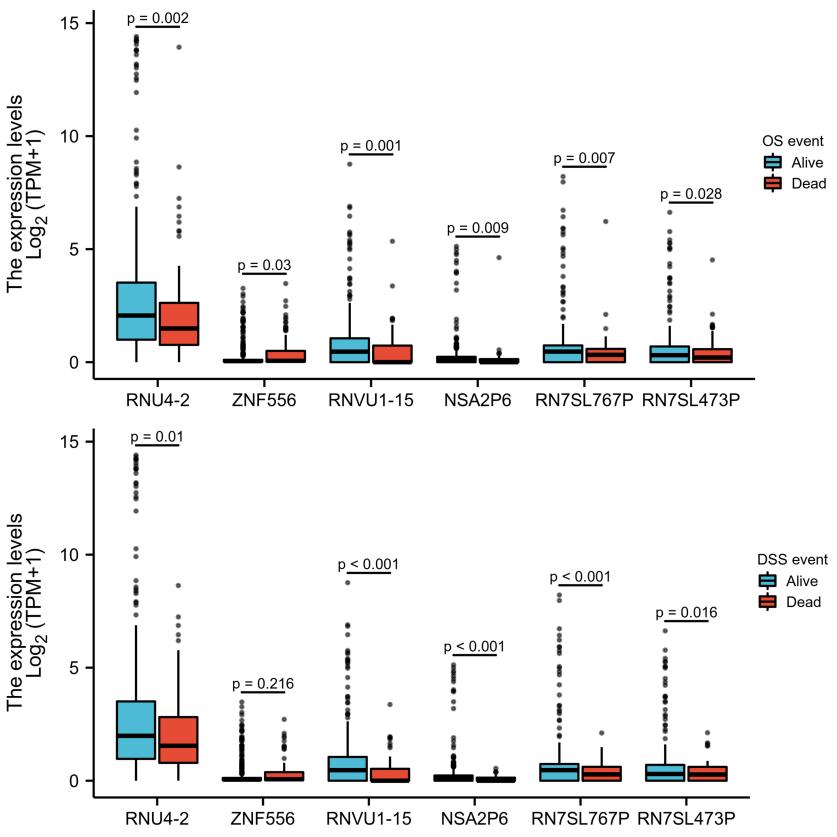


**Supplement Figure 1**: Expression of six genes in different groups.

Supplement: Supplementary Materials — Supplement Figure 1: expression of six genes in different groups. [file 4304361.f1.docx]
